# The CCAS-scale in hereditary ataxias: helpful on the group level, particularly in SCA3, but limited in individual patients

**DOI:** 10.1007/s00415-022-11071-5

**Published:** 2022-04-01

**Authors:** Andreas Thieme, Jennifer Faber, Patricia Sulzer, Kathrin Reetz, Imis Dogan, Miriam Barkhoff, Janna Krahe, Heike Jacobi, Julia-Elisabeth Aktories, Martina Minnerop, Saskia Elben, Raquel van der Veen, Johanna Müller, Giorgi Batsikadze, Jürgen Konczak, Matthis Synofzik, Sandra Roeske, Dagmar Timmann

**Affiliations:** 1 Department of Neurology and Center for Translational Neuro- and Behavioral Sciences (C-TNBS), Essen University Hospital, University of Duisburg-Essen, Hufelandstr. 55, 45147 Essen, Germany; 2 German Center for Neurodegenerative Diseases (DZNE) Bonn, Helmholtz Association, Venusberg-Campus 1/99, 53127 Bonn, Germany; 3 Department of Neurology, Bonn University Hospital, Rheinische Friedrich-Wilhelms University Bonn, Venusberg-Campus 1, 53127 Bonn, Germany; 4 Department of Neurodegenerative Diseases, Hertie-Institute for Clinical Brain Research and Center of Neurology, Eberhard-Karls University Tübingen, Hoppe-Seyler-Str. 3, 72076 Tübingen, Germany; 5 German Center for Neurodegenerative Diseases (DZNE) Tübingen, Helmholtz Association, Otfried-Müller-Str. 23, 72076 Tübingen, Germany; 6 JARA-BRAIN Institute, Molecular Neuroscience and Neuroimaging, Research Center Jülich, Wilhelm-Johnen-Str., 52425 Jülich, Germany; 7 Department of Neurology, Aachen University Hospital, Rheinisch-Westfälische Technische Hochschule (RWTH) Aachen, Pauwelstr. 30, 52074 Aachen, Germany; 8 Department of Neurology, Heidelberg University Hospital, Ruprecht-Karls University Heidelberg, Im Neuenheimer Feld 400, 69120 Heidelberg, Germany; 9 Department of Neurology, Center for Movement Disorders and Neuromodulation, Medical Faculty, Heinrich-Heine University Düsseldorf, Moorenstr. 5, 40225 Düsseldorf, Germany; 10 Institute of Clinical Neuroscience and Medical Psychology, Medical Faculty, Heinrich-Heine University Düsseldorf, Moorenstr. 5, 40225 Düsseldorf, Germany; 11 Institute of Neuroscience and Medicine (INM-1), Research Center Jülich, Wilhelm-Johnen-Str., 52425 Jülich, Germany; 12 School of Kinesiology, University of Minnesota, 400 Cooke Hall 1900 University Ave S E, Minneapolis, MN 55455 USA

**Keywords:** Cerebellum, SCA3, SCA6, FRDA, Bedside test

## Abstract

**Background:**

A brief bedside test has recently been introduced by Hoche et al. (Brain, 2018) to screen for the *Cerebellar Cognitive Affective Syndrome* (CCAS) in patients with cerebellar disease.

**Objective:**

This multicenter study tested the ability of the CCAS-Scale to diagnose CCAS in individual patients with common forms of hereditary ataxia.

**Methods:**

A German version of the CCAS-Scale was applied in 30 SCA3, 14 SCA6 and 20 FRDA patients, and 64 healthy participants matched for age, sex, and level of education. Based on original cut-off values, the number of failed test items was assessed, and CCAS was considered possible (one failed item), probable (two failed items) or definite (three failed items). In addition a total sum raw score was calculated.

**Results:**

On a group level, failed items were significantly higher and total sum scores were significantly lower in SCA3 patients compared to matched controls. SCA6 and FRDA patients performed numerically below controls, but respective group differences failed to reach significance. The ability of the CCAS-Scale to diagnose CCAS in individual patients was limited to severe cases failing three or more items. Milder cases failing one or two items showed a great overlap with the performance of controls exhibiting a substantial number of false-positive test results. The word fluency test items differentiated best between patients and controls.

**Conclusions:**

As a group, SCA3 patients performed below the level of SCA6 and FRDA patients, possibly reflecting additional cerebral involvement. Moreover, the application of the CCAS-Scale in its present form results in a high number of false-positive test results, that is identifying controls as patients, reducing its usefulness as a screening tool for CCAS in individual patients.

**Supplementary Information:**

The online version contains supplementary material available at 10.1007/s00415-022-11071-5.

## Introduction

Cerebellar disease results in well-known motor problems, including disordered limb coordination, dysarthria, impaired balance, and oculomotor dysfunction. In addition, cognitive and affective deficits have been reported in cerebellar disease, summarized under the term *Cerebellar Cognitive Affective Syndrome (CCAS)* [1]. Impairments of executive, language, and visuospatial abilities as well as affective dysfunction are considered the core features of CCAS [2, 3]. Recent functional and structural magnetic resonance imaging studies have mapped the motor, cognitive and emotional areas of the cerebellum (see [4–7] for reviews). The two main motor representations are located in the anterior cerebellar lobe (lobules I–V), with some extension into lobule VI and in lobule VIII in the posterior cerebellar lobe. Main non-motor areas cover most parts of the posterolateral cerebellar hemispheres (lobules VI, Crus I, Crus II, VIIB) [8].

Deficits in the core domains of CCAS have been observed in various types of cerebellar disease including spinocerebellar ataxia type 3 (SCA3), spinocerebellar ataxia type 6 (SCA6) and Friedreich’s ataxia (FRDA), three of the most common forms of hereditary ataxia [6]. Studies using more extended neuropsychological test batteries have reported that patients with SCA3, SCA6 and FRDA have difficulties in verbal fluency and verbal memory retrieval, working memory tasks, cognitive flexibility, abstract reasoning, and problem solving [9–20]. In addition, affective symptoms and deficits in social cognition have been observed [11, 21, 22]. Visuospatial disabilities are less clear. While some studies reported visuospatial deficits in SCA3 [23, 24] and FRDA [25, 26], other studies observed no deficits in SCA3 [11, 20, 27] and SCA6 [28]. Overall, the cognitive and affective abnormalities seen in patients with SCA3, SCA6 and FRDA conform to the pattern of CCAS. Cognitive dysfunction has been mapped to the posterolateral cerebellar hemisphere in SCA6 and FRDA patients [10, 29, 30]. In patients with SCA3, however, extracerebellar involvement may also contribute [31]. For example, progressive episodic memory loss has been reported in SCA3 which exceeds the core features of CCAS [18, 20, 27].

Despite the now well-established concept of CCAS in cerebellar disease, a standardized diagnostic tool to detect CCAS is missing. In 2018, a short and easily applicable bedside test, the CCAS-Scale, has been developed in American English. It has been validated in a group of adult patients with various cerebellar disorders [3]. Since its publication, the CCAS-Scale has been translated into different languages including German [32], and has already been in widespread use [33–37].

We asked the question whether the CCAS-Scale is able to diagnose CCAS in individual patients suffering from SCA3, SCA6 or FRDA.

## Methods

### Participants

Thirty patients with SCA3, 14 patients with SCA6 and 20 patients with FRDA were included in this study after giving written consent. An equal number of age-, sex- and education-matched healthy participants served as controls (Table 1). All participants were native German speakers. Data of 46 patients and 37 controls had been included in a preliminary study of our group [38]. Participants have been recruited at the University Hospitals of Aachen, Bonn, Düsseldorf, Essen, Heidelberg, and Tübingen. None of the participants suffered from psychiatric or neurological disorders other than SCA3, SCA6 or FRDA. Matched controls were selected out of a larger data pool (*n* = 180) which has been collected across study sites as part of an ongoing validation study. Controls who failed the item “delayed verbal recall” were not considered to rule out mild cognitive impairment. None of the participants took centrally acting drugs, except for a low-dose antidepressant in one SCA6 patient. The Scale for the Assessment and Rating of Ataxia (SARA) was used to rate the severity of the cerebellar motor syndrome [39]. Inventory of Non-Ataxia Signs (INAS) [40] score was available in 23 of the SCA3 patients (mean INAS count: 3.7 ± 2.2). One SCA3 patient showed extrapyramidal involvement (dystonia), 15 showed signs of pyramidal tract dysfunction, and 22 showed signs of polyneuropathy and/or dorsal column dysfunction. The study was approved by the local ethics committees and conforms to the Declaration of Helsinki.

**Table 1:** Patients’ and controls’ characteristics

	SCA3	SCA3 controls	SCA6	SCA6 controls	FRDA	FRDA controls
Number of males/females	12/18	8/22	12/2	11/3	11/9	12/8
Mean age ± SD (yrs)	50.5 ± 12.9	51.1 ± 13.1	62.3 ± 13.0	62.0 ± 12.7	40.2 ± 16.0	40.4 ± 17.2
	*U* = 443.5, * p* = 0.923		*U* = 96.0, * p* = 0.946		*U* = 199.0, * p* = 0.989	
Mean years of education ± SD	15.5 ± 3.3	15.5 ± 2.9	16.0 ± 3.0	15.6 ± 3.3	16.2 ± 3.0	16.2 ± 3.1
	*U* = 431.0, * p* = 0.777		*U* = 88.0, * p* = 0.667		*U* = 192.5, * p* = 0.841	
Mean age at disease onset ± SD (yrs)	39.5 ± 11.7	–	53.0 ± 12.4	–	20.7 ± 12.3	–
Mean disease duration ± SD (yrs)	11.0 ± 8.2	–	10.0 ± 11.8	–	19.5 ± 10.6	–
Mean SARA score ± SD	12.9 ± 7.5	–	10.8 ± 5.9		20.4 ± 7.7	
Mean repeat length of expanded alleles ± SD (number of patients with known repeat length)	69 ± 4 (n = 23)	–	22 ± 2 (n = 11)	–	Longer allele: 730 ± 225; Shorter allele: 480 ± 245 (n = 19)	–

Statistics: Two-sided Mann–Whitney *U* tests were applied to test for age and level of education differences between the patient and corresponding control groups

*SCA3* spinocerebellar ataxia type 3, *SCA6* spinocerebellar ataxia type 6, *FRDA* Friedreich’s ataxia, *SD* standard deviation, *SARA* scale for the assessment and rating of ataxia, *yrs* years, *n* number

### Cerebellar cognitive affective syndrome scale (CCAS-Scale)

All participants performed version A of the German CCAS-Scale [32]. The CCAS-Scale consists of 12 items. Performance in ten items is scored: semantic and phonematic word fluency, category switching, digit span forward and backward, cube drawing, delayed verbal recall, similarities, go/ no-go, and affect (for details see [3] and [32]). Each test item has a raw score. Based on item-specific thresholds introduced in the original US-American validation study an item is either passed or failed. According to Hoche et al. [3], the presence of CCAS is considered possible if one item is failed, probable if two items are failed, and definite if three or more items are failed. Additionally, a total sum score can be calculated (range: 0–120) by summation of the single items’ raw scores.

### Statistics

Results from a Shapiro–Wilk Test showed that the data were not normally distributed. The number of failed test items, the total sum raw scores and raw scores of single test items were compared using estimation statistics focusing on effect size rather than solely on significance testing (https://www.estimationstats.com). Permutated *t* tests were calculated. For each permutation *p* value, 5000 reshuffles were performed. The null hypothesis was rejected for a *p* value < 0.05. The permutated *t* test is robust to non-normal distributions [41].

Selectivity and sensitivity were assessed using the original cut-off values for individual test items, and the three pass/fail criteria introduced by Hoche et al. [3]. To assess selectivity the percentage of true-negatives was calculated, that is the percentage of controls which have been correctly identified as controls (number of controls identified as controls/ true number of controls in the sample*100). To assess sensitivity the percentage of true-positives was calculated, that is the percentage of patients which have been correctly identified as patients (number of patients identified as patients/ true number of patients in the sample*100) [38].

The percentage of patients and controls being categorized as CCAS “absent”, “possible”, “probable” and “definite” was calculated. In addition, the percentage of patients and controls failing individual test items was calculated. Fisher’s exact test was used to assess group differences, because sample sizes were small.

A receiver operating curve (ROC) analysis was performed graphing the true-positive versus the false-negative rate considering the number of failed test items, total sum score and single test items’ raw scores. An area under the curve (AUC) of < 0.5 indicates that a test does not exceed chance level in discriminating patients from controls, while an AUC of 1 reflects a perfect relationship between the true-positive and the false-negative rate [42]. In accordance with the previous literature, an AUC between 0.5 and 0.7 was considered poor, between 0.7 and 0.8 was considered good and an AUC of > 0.8 was considered excellent [43–45]. Optimal cut-offs for total failed items and total sum raw score were calculated using Youden’s Index (YI = sensitivity for a specific cut-off value + selectivity for that cut-off value − 1). YI indicates the cut-off for which the relationship between true-positives (that is, sensitivity) and false-negatives (that is, 100% − selectivity) is optimal [36, 46].

Finally, possible relationships between the scores (that is, the number of failed test items or the total raw sum score) and age, years of education, and (in patients) the SARA score were examined using multiple linear regression analyses. Additionally, the age at disease onset and the disease duration as well as trinucleotide repeat length were included as independent variables to calculate Spearman’s rank correlation coefficients. The latter variables were not included in the linear regression analyses because these variables showed a high correlation with the SARA score or the age of the participants (see correlation analyses in supplements for details).

## Results

### Total failed test items

**Fig. 1:**
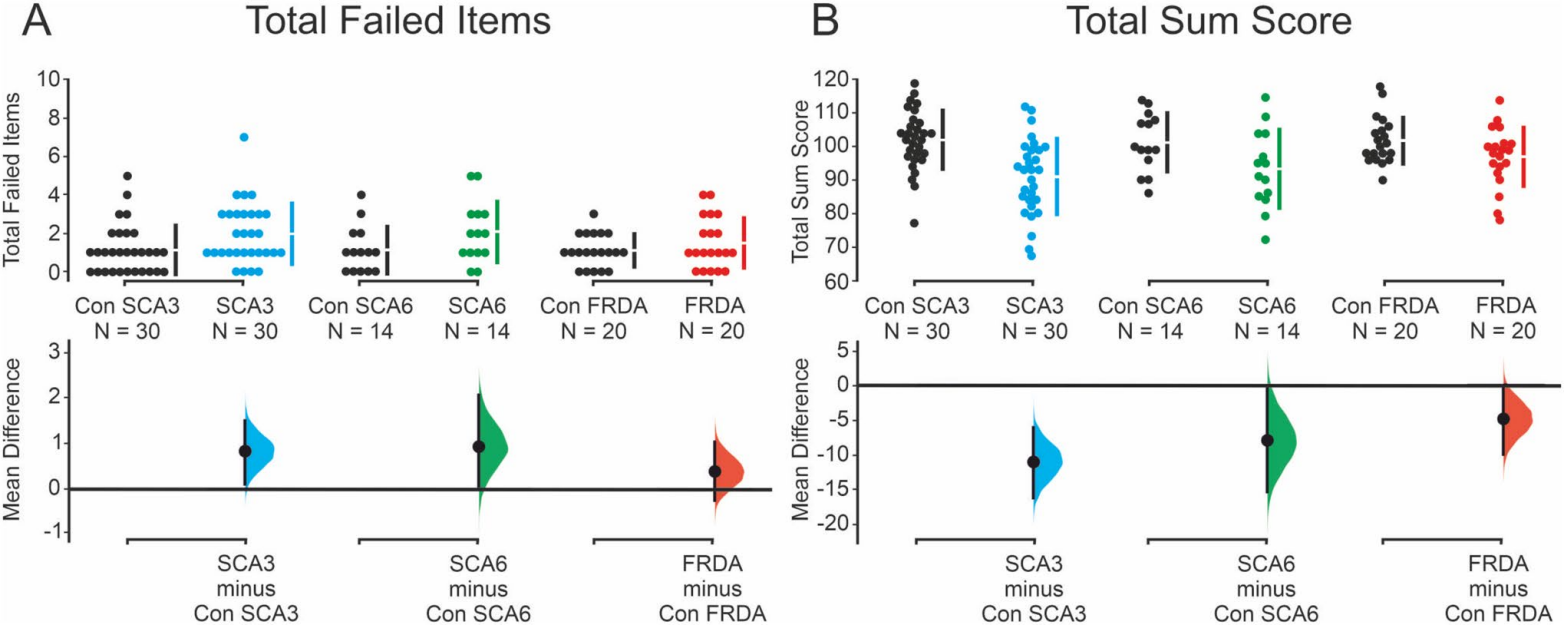
Total failed test items and total sum score. The total number of failed test items (**A**) and the total sum score (**B**) are shown for SCA3 (blue), SCA6 (green) and FRDA patients (red) and their respective matched controls (black). Upper panels: Each circle represents one participant. Discontinuous bars show means and standard deviations. Lower panels: Mean differences are plotted as a bootstrap sampling distribution. Dots represent mean difference, and 95% confidence intervals are indicated by the ends of the vertical error bars

Patients with SCA3 failed on average more test items (2.0 ± 1.6) compared to matched controls (1.1 ± 1.3). The number of failed test items was also numerically higher in SCA6 patients, and to lesser degree in FRDA patients, compared to controls (SCA6: 2.1 ± 1.6 vs. SCA6 controls: 1.1 ± 1.2; FRDA: 1.5 ± 1.3 vs. FRDA controls: 1.1 ± 0.9). The difference between SCA3 patients and SCA3 controls was significant [unpaired mean difference (MD): 0.8; 95% confidence interval (CI), lower bound, upper bound: 0.1, 1.5; *p* = 0.024]. The comparison between SCA6 and SCA6 controls (MD: 0.9; CI 0.0, 2.1; *p* = 0.074), and between FRDA and FRDA controls (MD: 0.4; CI − 0.3, 1.1, *p* = 0.204; two-sided permutation t test; Fig. 1A) failed significance.

### Total sum score

Patients with SCA3 reached a lower total sum score (90.9 ± 11.4) compared to SCA3 controls (102.0 ± 8.9). Total sum score was also lower in SCA6 patients (93.3 ± 11.9) compared to SCA6 controls (101.3 ± 8.6), and FRDA patients (96.9 ± 8.9) compared to FRDA controls (102.0 ± 7.0). The difference between SCA3 patients and SCA3 controls was significant (MD: − 11.1; CI − 16.3, − 6.1; *p* < 0.001). Comparisons of FRDA (MD: − 5.0; CI − 10.1, − 0.4; *p* = 0.051) and SCA6 patients and matched controls (MD: − 8.0; CI − 15.4, − 0.3; *p* = 0.057; two-sided permutation *t* test; Fig. 1B) failed significance.

### Single test items

Based on pass/fail criteria using the cut-offs introduced by Hoche et al. [3], the items “phonematic fluency” and “category switching” showed the largest differences between the three groups of patients and their matched controls (Table 2; Fig. 1 supplements). Fisher’s exact test revealed significant differences comparing the number of SCA3 patients and SCA3 controls failing “phonematic fluency”, and the number of FRDA patients and FRDA controls failing “category switching”. “Semantic fluency” was close to significance comparing SCA3 patients and controls. All other comparisons were not significant (all *p* values > 0.1).

**Table 2:** Performance on single test items: percentage of participants failing single test items and raw scores

Test Item	SCA3	SCA3 controls	SCA6	SCA 6 controls	FRDA	FRDA controls
Percentage of participants failing on single test items						
Semantic fluency	17%	0%	0%	0%	10%	0%
	*p* = 0.052		–		*p* = 0.487	
Phonematic fluency	**57%**	**27%**	43%	29%	40%	30%
	*p* = 0.035*		*p* = 0.695		*p* = 0.741	
Category switching	37%	17%	43%	14%	**45%**	**10%**
	*p* = 0.143		*p* = 0.209		*p* = 0.031*	
Digit span forward	20%	27%	36%	36%	25%	20%
	*p* = 0.761		*p* = 1.0		*p* = 1.0	
Digit span backward	20%	17%	21%	7%	10%	15%
	*p* = 1.0		*p* = 0.596		*p* = 1.0	
Cube drawing	7%	7%	0%	14%	5%	10%
	*p* = 1.0		*p* = 0.481		*p* = 1.0	
Verbal recall	10%	0%	21%	0%	5%	0%
	*p* = 0.237		*p* = 0.222		*p* = 1.0	
Similarities	10%	0%	0%	7%	0%	5%
	*p* = 0.237		*p* = 1.0		*p* = 1.0	
Go/no-go	10%	27%	29%	7%	5%	20%
	*p* = 0.181		*p* = 0.326		*p* = 0.342	
Affect	7%	0%	14%	0%	10%	0%
	*p* = 0.492		*p* = 0.481		*p* = 0.487	
Raw scores on single test items						
Semantic fluency	**19.8 ± 4.6**	**23.8 ± 3.1**	21.9 ± 3.6	23.5 ± 3.5	**21.1 ± 3.9**	**24.3 ± 2.5**
	MD: − 4.0; CI − 6.0, − 2.1; *p* < 0.001**		MD: − 1.6; CI − 4.0, 1.0; *p* = 0.203		MD: − 3.2; CI − 5.3, − 1.3; *p* = 0.002*	
Phonematic fluency	**8.8 ± 4.1**	12.4 ± 3.9	9.9 ± 3.7	12.5 ± 4.5	11.6 ± 4.6	11.2 ± 4.7
	MD: − 3.6; CI − 5.7, − 1.7; *p* < 0.001**		MD: − 2.6; CI − 5.5, 0.4; *p* = 0.092		MD: 0.4, CI − 2.4, 3.2; *p* = 0.076	
Category switching	**10.8 ± 3.5**	12.8 ± 2.8	10.2 ± 4.4	12.6 ± 2.9	**10.2 ± 2.8**	**12.7 ± 2.6**
	MD: − 2.0; CI − 3.6, − 0.4;* p* = 0.022*		MD: − 2.4; CI − 5.2, 0.1;* p* = 0.089		MD: − 2.6; CI − 4.1, − 0.8;* p* < 0.004*	
Digit span forward	6.2 ± 1.1	6.2 ± 1.1	6.2 ± 1.1	5.9 ± 1.4	6.3 ± 1.1	6.6 ± 1.2
	MD: 0.0; CI − 0.6, 0.5;* p* = 0.905		MD: 0.4; CI − 0.5, 1.3;* p* = 0.374		MD: − 0.3; CI − 1.1, 0.4;* p* = 0.347	
Digit span backward	**3.9 ± 0.9**	4.4 ± 1.0	4.2 ± 1.1	4.5 ± 0.9	4.4 ± 0.9	4.5 ± 1.0
	MD: − 0.5; CI − 1.0, − 0.1;* p* = 0.035*		MD: − 0.3; CI − 1.1, 0.4;* p* = 0.355		MD: − 0.1; CI − 0.7, 0.5;* p* = 0.74	
Cube drawing	13.9 ± 1.7	13.8 ± 1.6	14.4 ± 1.3	13.8 ± 1.7	14.7 ± 1.1	13.8 ± 1.8
	MD: 0.0; CI − 0.9, 0.8;* p* = 0.881		MD: 0.6; CI − 0.6, 1.6;* p* = 0.21		MD: 0.9; CI − 0.1, 1.8;* p* = 0.067	
Verbal recall	13.2 ± 2.1	13.5 ± 1.2	12.2 ± 3.1	13.1 ± 1.4	13.5 ± 1.8	13.9 ± 1.2
	MD: − 0.3; CI − 1.3, 0.4;* p* = 0.403		MD: − 0.9; CI − 3.1, 0.6;* p* = 0.332		MD: − 0.5; CI − 1.5, 0.4;* p* = 0.314	
Similarities	7.4 ± 0.9	7.6 ± 0.5	7.6 ± 0.6	7.6 ± 0.6	7.8 ± 0.4	7.8 ± 0.6
	MD: − 0.2; CI − 0.6, 0.2;* p* = 0.283		MD: 0.1; CI − 0.4, 0.6;* p* = 0.552		MD: 0.1; CI − 0.3, 0.4;* p* = 0.52	
Go/no-go	1.4 ± 0.7	1.1 ± 0.8	1.1 ± 0.9	1.5 ± 0.7	**1.8 ± 0.5**	**1.3 ± 0.8**
	MD: 0.3; CI − 0.2, 0.6;* p* = 0.125		MD: − 0.4; CI − 0.9, 0.1;* p* = 0.142		MD: 0.5; CI 0.1, 0.9;* p* = 0.01*	
Affect	5.6 ± 1.0	5.9 ± 0.3	5.5 ± 1.3	5.9 ± 0.3	5.7 ± 0.7	5.9 ± 0.4
	MD: − 0.3; CI − 0.9, − 0.1;* p* = 0.034*		MD: − 0.4; CI − 1.6, 0.0;* p* = 0.229		MD: − 0.2; CI − 0.6, 0.1;* p* = 0.145	

Statistics: two-sided Fisher’s exact test was applied to compare the patient and corresponding control groups regarding the percentage of participants who failed on specific test items. For the comparison of raw scores of single test items a two-sided permutated t test was applied. Significant results are indicated in bold font and by asterisks

*SCA3* spinocerebellar ataxia type 3, *SCA6* spinocerebellar ataxia type 6, *FRDA* Friedreich’s ataxia, *MD* unpaired mean difference, *CI* confidence interval (lower bound, upper bound)

**Fig. 2:**
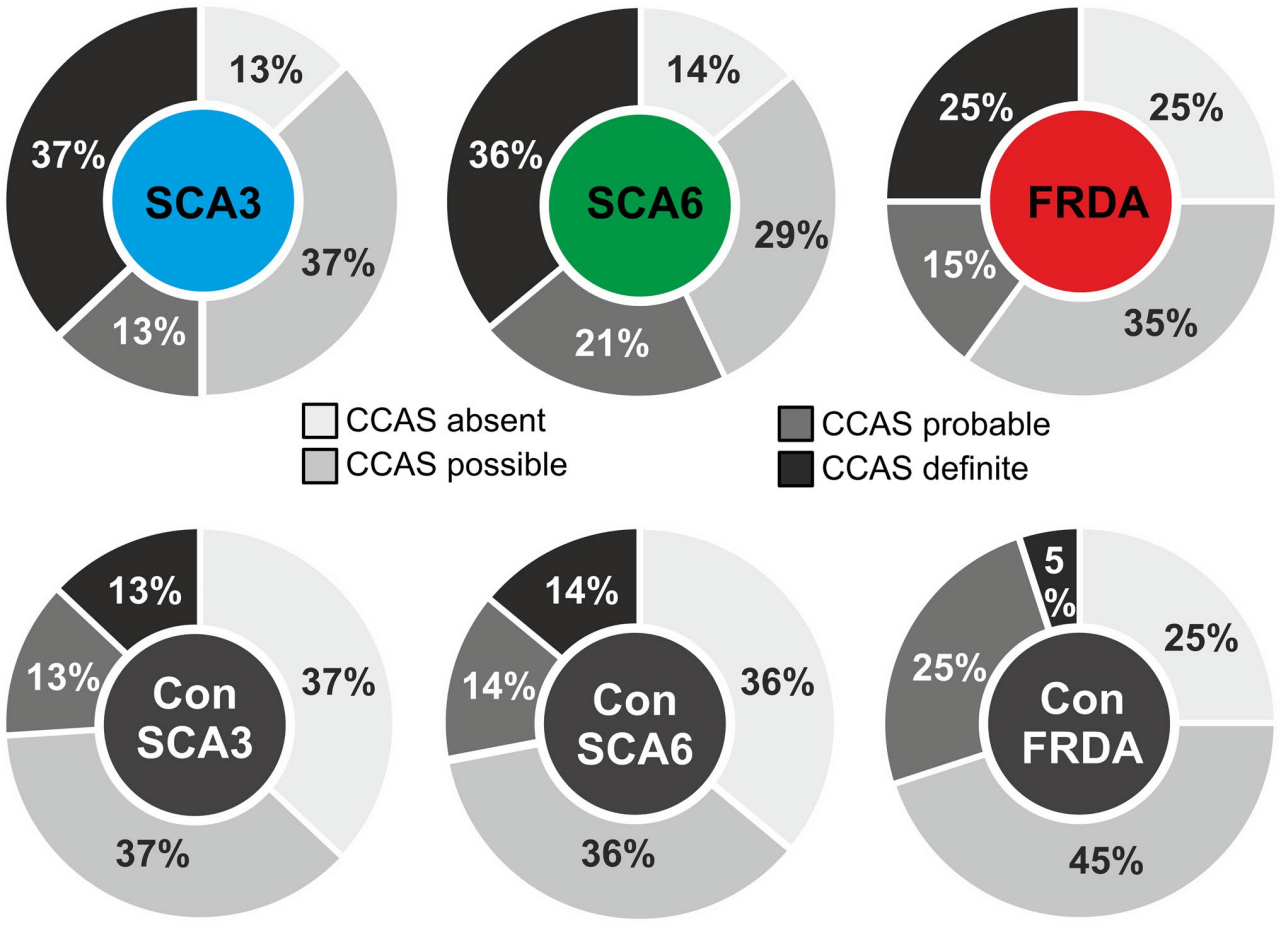
Categorization as “CCAS absent/possible/probable/definite”. Percentage of patients and matched controls categorized as CCAS absent (no item failed), possible (one failed item), probable (two failed items) or definite (three or more failed test items) based on original cut-off values^3^

Based on raw scores of single test items, the items “semantic” and “phonematic fluency”, and “category switching” showed the largest differences between patients and controls (Tab. 2; Fig. 2 supplements). Group differences were significant comparing SCA3 patients and SCA3 controls for raw scores of “semantic” and “phonematic fluency”, “category switching”, “digit span backward” and “affect” (*p* values < 0.05, two-sided permutation t test). FRDA patients performed significantly below FRDA controls considering raw scores of “semantic fluency” and “category switching” (*p* values < 0.01). FRDA patients performed significantly higher on the “go/ no-go” task than FRDA controls. SCA6 patients’ did not differ significantly from controls’ raw scores in any test item (*p* values > 0.08).

### Categorization as “CCAS possible/probable/definite”

Based on the number of failed test items, a diagnosis of CCAS was considered possible in 37% of the SCA3 patients, probable in 13% and definite in 37%. In an equal percentage of SCA3 controls, CCAS was considered possible (37%) or probable (13%). CCAS was considered definite in 13% of SCA3 controls. A similar pattern was observed in SCA6 and FRDA patients and their matched controls: SCA6: 29/21/36% (CCAS possible/probable/definite), SCA6 controls: 36/14/14%, FRDA: 35/15/25%, FRDA controls: 45/25/5% (Fig. 2). Thus, a similar percentage of patients and matched controls presented with one or two failed test items. Three or more failed test items were found on average more often in patients compared to controls and were present more often in SCA3 patients compared to SCA6 and FRDA patients. None of these differences reached significance (two-sided Fisher’s exact test; all *p* values > 0.07).

The CCAS-Scale showed good to excellent values for selectivity (i.e. the ability to distinguish between patients and controls, or in other words preventing controls from being diagnosed as patients) only for “CCAS definite”, but not for the categories “CCAS possible” or “probable”: 37/74/87% true-negatives in SCA3 controls, 36/72/86% in SCA6 controls, and 25/70/95% in FRDA controls. Sensitivity (that is true-positives) for possible/probable/definite CCAS was 87/50/37% in SCA3 patients, 86/57/36% in SCA6 patients, and 75/40/25% in FRDA patients.

### Receiver operating curves (ROC)

**Fig. 3:**
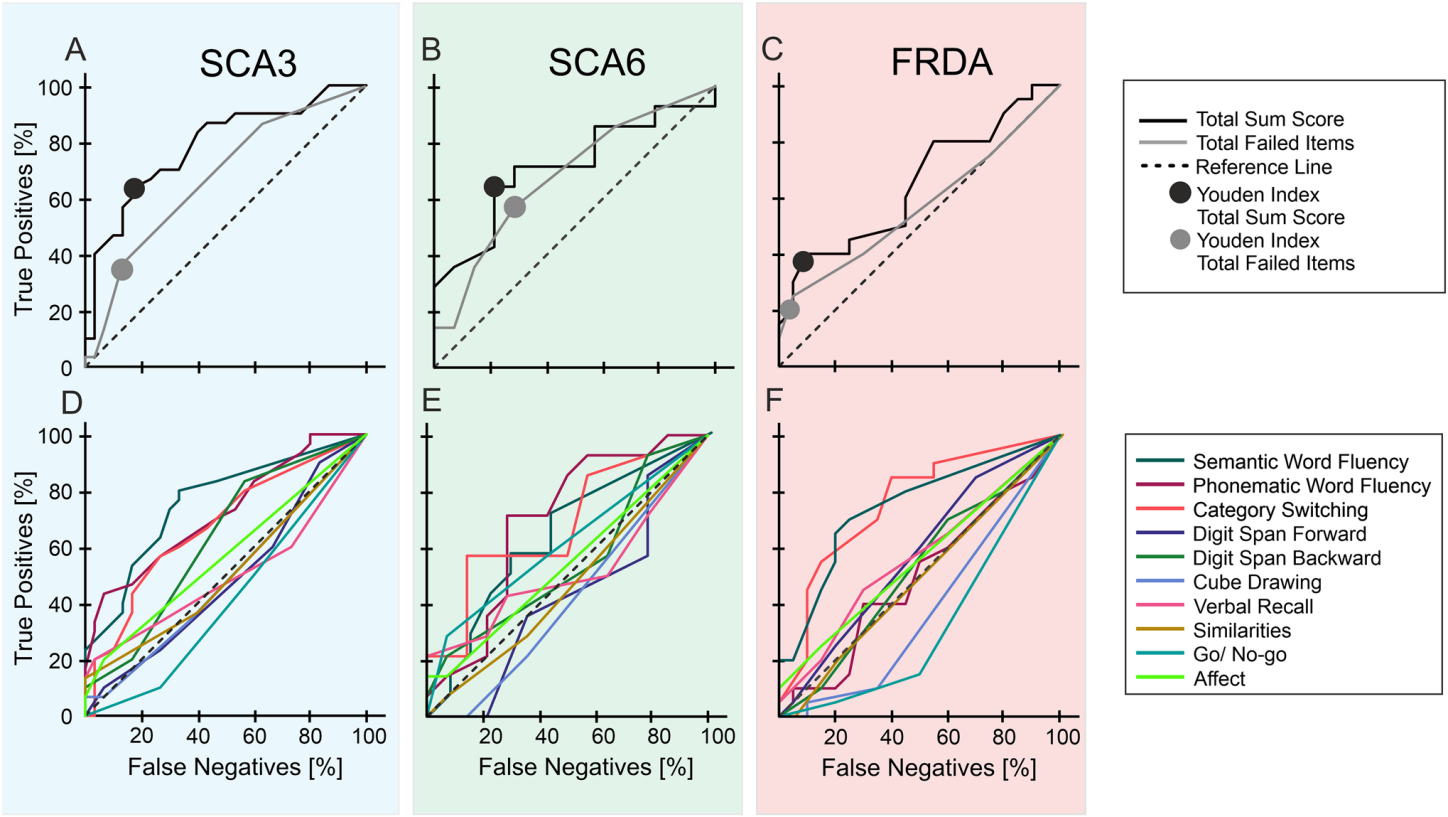
Receiver operating curves (ROC) for all group comparisons. ROC of total failed test items and total sum score (**A**–**C**) as well as single test item raw scores (**D**–**F**) are shown for SCA3 patients and SCA3 controls (blue background), SCA6 patients and SCA6 controls (green background) and FRDA patients and FRDA controls (red background). For total failed test items and total sum score the Youden Index is given (**A**–**C**)

In the SCA3 and SCA3 control groups, ROC analysis revealed poor discriminative ability for the number of failed items [AUC ± standard error (SE): 0.67 ± 0.07, *p* = 0.023], which was improved considering the total sum score (AUC ± SE: 0.79 ± 0.06, *p* < 0.001; Fig. 3A). In the SCA6 and SCA6 control group, ROC analysis was poor for total failed items (AUC ± SE: 0.68 ± 0.10, *p* = 0.103), and showed some improvement considering total sum score (AUC ± SE: 0.71 ± 0.10, *p* = 0.057; Fig. 3B). The differentiation between FRDA patients and FRDA controls was also poor for the number of failed items (AUC ± SE: 0.57 ± 0.09, *p* = 0.465), which was improved, but still poor for total sum score (AUC ± SE: 0.65 ± 0.09, *p* = 0.110; Fig. 3C).

Regarding single test item raw scores in SCA3 patients and controls, ROC analyses revealed good discriminative ability for “semantic” (AUC ± SE: 0.76 ± 0.06, *p* = 0.001) and “phonematic fluency” (AUC ± SE: 0.72 ± 0.07, *p* = 0.003). For all other items, the differentiation between SCA3 patients and SCA3 controls was poor (AUC < 0.67; Fig. 3D).

In SCA6 patients and controls, ROC analyses revealed poor discrimination for all test items’ raw scores (AUC < 0.7), with “phonematic fluency” (AUC ± SE: 0.69 ± 0.10, *p* = 0.081) and “category switching” (AUC ± SE: 0.68 ± 0.10, *p* = 0.098; Fig. 3E) performing best.

Regarding single test item raw scores in FRDA patients and controls, ROC analyses revealed good discriminative ability for “semantic fluency” (AUC ± SE: 0.75 ± 0.08, *p* = 0.008) and “category switching” (AUC ± SE: 0.76 ± 0.08, *p* = 0.006). Differentiation between FRDA patients and FRDA controls was poor for all other test items (AUC < 0.6; Fig. 3F).

For total failed test items and total sum score, optimal cut-offs were determined using Youden’s Index (indicated by circles in Fig. 3A–C). Three failed items distinguished best between FRDA and SCA3 patients and their respective controls and two failed items distinguished SCA6 patients best from their matched controls. As outlined above, these cut-off values showed good to excellent values for selectivity in SCA3 controls (87% true-negatives) and FRDA controls (95% true-negatives), but only moderate values for selectivity in SCA6 controls (72% true-negatives). Sensitivity for these cut-offs (that is true-positives) was poor to moderate: 37% in SCA3 patients, 57% in SCA6 patients, and 25% in FRDA patients.

For total sum score, the optimal cut-off was 95 in all groups. These cut-off values improved selectivity in controls to some extent (true-negatives: SCA3/SCA6/FRDA controls: 83/79/90%), and sensitivity in particular in SCA3 and FRDA patients (true-positives: SCA3/SCA6/FRDA patients: 63/64/40%).

### Linear regression analyses

In SCA3 patients, age, years of education, and SARA score did not significantly predict the number of failed items [*F* (3, 26) = 2.265, *p* = 0.105, *R*^2^ = 0.207], but significantly predicted the total sum score [*F* (3, 26) = 7.382, *p* = 0.001, *R*^2^ = 0.460]. Years of education (*p* = 0.021) and the SARA score (*p* = 0.001), but not age (*p* = 0.319) added significantly to the prediction. SCA3 patient’s total sum score was equal to: 77.150 + 0.166 * age [years] + 1.247 * education [years] – 1.076 * SARA score.

In SCA6 and FRDA patients, age, years of education, and SARA score did not significantly predict the number of failed test items [SCA6: *F* (3, 9) = 1.754, *p* = 0.226, *R*^2^ = 0.369; FRDA:* F* (3, 15) = 0.429,* p* = 0.735,* R*^2^ = 0.079] or the total sum score [*F* (3, 9) = 0.044, *p* = 0.987, *R*^2^ = 0.014; FRDA: *F* (3, 15) = 1.338, *p* = 0.299, *R*^2^ = 0.211].

In the group of all controls, multiple regression revealed that age and years of education significantly predicted both the number of failed test items [*F* (2, 61) = 4.793, *p* = 0.012, *R*^2^ = 0.136] and the total sum score [*F* (2, 61) = 6.126, *p* = 0.004, *R*^2^ = 0.167]. The two variables added significantly to the prediction of the total sum score and age added significantly to the prediction of the total number of failed items (*p* values < 0.05), while the contribution of years of education was close to significance (*p* = 0.083). Control’s predicted total number of failed items was equal to: 1.335 + 0.02 * age [years] – 0.079 * education [years]. Their predicted total sum score was equal to: 99.512 − 0.161 * age [years] + 0.660 * education [years].

### Correlation analyses

In SCA3 patients, SARA score was positively correlated with the number of failed items (*R* = 0.407, *p* = 0.026; Fig. S3A, supplements) and inversely correlated with total sum score (*R* = − 0.594, *p* = 0.001; Fig. S3B, supplements). The number of failed items (*R* = 0.384, *p* = 0.036) and total sum score (*R* = − 0.443, *p* = 0.014) correlated with disease duration.

In FRDA and SCA6 patients, the number of failed items and total sum score did not correlate with SARA score or disease duration.

When pooling all controls, age correlated with the number of failed items (*R* = 0.320, *p* = 0.01) and total sum score (*R* = − 0.346, *p* = 0.005). Correlations between years of education and failed items (*R* = − 0.246, *p* = 0.050) and total sum score (*R* = 0.237, *p* = 0.059) were close to significance. All other correlations were not significant (*p* values > 0.05; Tab. S1, supplements).

## Discussion

This study investigated the usefulness of the recently introduced CCAS-Scale to screen for cognitive abnormalities in individual patients with SCA3, SCA6 or FRDA. On a group level, SCA3 patients performed significantly poorer than controls when using the CCAS-Scale, but not SCA6 and FRDA patients. Moreover, the performance of patients with either of the three types of ataxia showed substantial overlap with the performance of controls, limiting the value of the CCAS-Scale to screen for CCAS on the level of individual patients.

### CCAS-Scale reveals significant abnormalities in SCA3 on a group level, but not in SCA6 and FRDA

The number of failed CCAS-Scale test items was significantly increased, and the total sum score was significantly reduced in SCA3 patients compared to matched controls confirming recent findings [34]. Findings in SCA6 and FRDA patients, however, were not significantly different from controls, although the number of failed items was numerically higher, and the total sum score was numerically lower. Cognitive deficits may be most pronounced in SCA3 patients for at least two reasons. First, the involvement of cortical and subcortical cerebral regions may play a role [18, 47]: in SCA3 patients, a correlation between episodic and working memory deficits and a reduction of grey matter density has been observed in the cerebellum, as well as temporal, frontal, and parietal regions and the insula [18]. Likewise, using single-proton emission computed tomography, associations between the perfusion of the parahippocampal gyrus, basal ganglia and thalamus and impaired performance on visuospatial and executive tests have been reported in SCA3 patients [23]. Second, the dentate nuclei are strongly affected in SCA3. Cerebellar nuclei are the main output structures of the cerebellum, and similar to the cerebellar cortex there is compartmentalization of the dentate nuclei in motor and cognitive areas [48, 49]. Patients with focal lesions of the dentate nuclei performed worse on cognitive testing than patients with cerebellar lesions sparing the dentate nuclei [25]. However, involvement of the dentate nuclei alone cannot explain group differences because atrophy of the dentate nucleus is also a hallmark of FRDA [51–53].

Lack of significant differences comparing SCA6 patients and controls agree with reports in the literature that cognitive deficits in SCA6 are commonly mild [9, 28]. SCA6 is a purer form of cerebellar degeneration, and cognitive deficits are primarily attributed to atrophy of cognitive areas within the cerebellar cortex [12].

Lack of significant group differences in FRDA patients are at variance with previous reports using more extensive neuropsychological test batteries [16, 25, 54]. Furthermore, Naeije and colleagues reported more failed CCAS-Scale test items and a lower CCAS-Scale total sum score in FRDA patients compared to the present study [37]. A higher percentage of late-onset FDRA patients might explain the milder cognitive deficits in the present study population [26]. Early disease onset might interfere with neurodevelopmental processes and, therefore, lead to more severe cognitive deficits [55].

In patients with SCA3, severity of CCAS, quantified by the CCAS-Scale, correlated with severity of the cerebellar motor syndrome, quantified by the Scale for Assessment and Rating of Ataxia (SARA), confirming recent findings by Maas et al. [34]. In patients with SCA6 and FRDA, we did not observe such correlations. Naeije and colleagues, however, reported a correlation between CCAS-Scale and SARA scores in patients with FRDA [37]. A wider range of cognitive and motor dysfunction in the Naeije et al. study may explain the difference. CCAS, on the other hand, may not always parallel the cerebellar motor syndrome, because the degree of degeneration may vary in cognitive and motor cerebellar areas (see [56] for review). As expected, abnormalities on the CCAS-Scale have been mapped to the posterolateral cerebellum in patients with chronic cerebellar stroke [36]. With respect to SCA3 patients, the involvement of cerebral areas may also play a role. Brain MRI scans, however, were not available in the present study to confirm these assumptions.

In summary, on the group level, the CCAS-Scale was able to detect cognitive involvement in SCA3, but not in SCA6 and FRDA patients. These findings are not explained by disease duration or disease severity, because these were worst in FRDA patients. Despite small group size and thereby less statistical power in the SCA6 and FRDA patient groups, the present findings indicate that the CCAS-Scale may have less diagnostic power in more pure forms of cerebellar degeneration and predominantly sensory ataxias compared to ataxias with extracerebellar, cerebral involvement. Lack of significant group differences comparing CCAS-Scale performance in patients with more pure cerebellar phenotypes and matched controls have also been reported by others [57].

### The ability of the CCAS-Scale to diagnose CCAS in individual cases is limited

There was significant overlap comparing the three patient samples and matched controls with many controls being diagnosed with possible (one item failed) or probable (two items failed) CCAS. Hence, selectivity was poor for “CCAS possible” (FRDA/SCA3/SCA6 controls: 25/37/36%) and moderate for “CCAS probable” (FRDA/SCA3/SCA6 controls: 70/74/72%). Selectivity was good only for “CCAS definite” (three or more items failed): FRDA/SCA3/SCA6 controls: 95/87/86%. The present findings are in line with two other studies including controls (selectivity for CCAS possible/probable/definite: Maas et al. [34]: 17/56/78%; Chirino-Pérez et al. [36]: 32/68/91%). While selectivity was good for “CCAS definite”, sensitivity (the ability to detect true-positives) was poor (FRDA/ SCA3/SCA6: 25/37/36%). Likewise, sensitivity (for “CCAS definite”) was moderate in previous studies testing SCA3 patients: 55% [34], FRDA patients: 63% [37], and chronic cerebellar strokes: 54% [36].

One reason for poor selectivity may be that neither the original cut-off values for individual test items, nor the number of total failed test items indicative of CCAS are corrected for age, education, or sex [3]. Similar to our previous study [38], we found that the number of failed test items and the total sum score were age and education dependent. Education dependency has also been reported by others [33, 36]. In the present study, however, patients’ subgroups were compared with matched controls. Therefore, age, education and sex effects are unlikely to explain the poor differentiation between patients and controls alone.

Some of the test items appear to better differentiate between patients and controls than others. For example, controls failed similarly often on the digit span tasks as patients. Preserved digit span in cerebellar disease has also been reported by others [9, 10, 28, 31, 34, 58]. The word fluency tasks differentiated best between patients and controls. Differences were most prominent considering raw scores. Compared to controls, raw scores were significantly lower for semantic and phonematic fluency tasks as well as category switching in SCA3 patients, and for semantic fluency and category switching in FRDA patients. In SCA6 patients, raw scores were numerically lower. Likewise, total sum scores appeared to be better suited for differentiation between patients and controls than the number of failed test items, a finding which has also been observed by Maas et al. [34] in SCA3 patients.

Van Overwalle and colleagues, who also observed poor differentiation between cerebellar patients and controls based on CCAS-Scale performance, reported that a more sensitive screening test is a test of social cognition [57]. In the future, it will be of interest to study whether one or two word fluency tasks or a test of social cognition suffice to screen for the presence of CCAS in individual patients. In clinical trials and patients with more focal cerebellar disease, however, it will be of interest to test for deficits in all of the cognitive and affective domains included in the original scale. Although degenerative cerebellar disease is usually more widespread, reduction to word fluency or social cognition tasks has the risk to miss patients with lesions more restricted to specific cerebellar areas, for example emotional areas of the cerebellum, given the small grained functional compartmentalization [4, 8].

## Conclusions

The CCAS-Scale showed more pronounced deficits in SCA3 patients compared to SCA6 and FRDA patients, likely because of cerebral involvement in SCA3. The word fluency tasks differentiated best between patients and controls. Although patients performed below controls on a group level, particularly in SCA3, selectivity of the CCAS-Scale was low resulting in a high percentage of false-positives test results. To improve the diagnostic value of the CCAS-Scale, adjustments of cut-off values, other weighting, exclusion of items or introduction of additional items may be necessary.

## Supplementary Information

Below is the link to the electronic supplementary material.

Supplementary file1 (DOCX 1182 kb)

## Data Availability

Data underlying the statistics and the figures is available from the corresponding author upon request.

## Declarations

### Study registration

The study has prospectively been registered at the German Clinical Study Register (https://www.drks.de; DRKS-ID: DRKS00016854).
