# Supplementary material for: The CCAS-scale in hereditary ataxias: helpful on the group level, particularly in SCA3, but limited in individual patients
Source: J Neurol. 2022 Apr 1;269(8):4363–74. doi: 10.1007/s00415-022-11071-5 (PMC9293809; doi:10.1007/s00415-022-11071-5)
Supplement: Supplementary file 1 — Supplementary file1 (DOCX 1182 kb) [file 415_2022_11071_MOESM1_ESM.docx]

**Supplementary Materials to the article**

**“The CCAS-Scale in hereditary ataxias: Helpful on the group level but limited in individual patients”**

Andreas Thieme, MD; Jennifer Faber, MD; Patricia Sulzer, M. Sc.; Kathrin Reetz, MD; Imis Dogan, PhD; Miriam Barkhoff, B. Sc.; Janna Krahe, M. Sc.; Heike Jacobi, MD; Julia-Elisabeth Aktories, MD; Martina Minnerop, MD; Saskia Elben, MD; Raquel van der Veen, MD; Johanna Müller, MD; Giorgi Batsikadze, PhD; Jürgen Konczak, PhD; Matthis Synofzik, MD; Sandra Roeske, PhD; Dagmar Timmann, MD

(Journal of Neurology)

**Corresponding author**

Andreas Gustafsson Thieme, MD (ORCID: 0000-0001-6221-8601)

Essen University Hospital, Department of Neurology and Center for Translational Neuro- and Behavioral Sciences (C-TNBS), Hufelandstraße 55, 45147 Essen, Germany; Phone: +49 201 723 2180; E-Mail: [andreas.thieme@uk-essen.de](mailto:andreas.thieme@uk-essen.de)


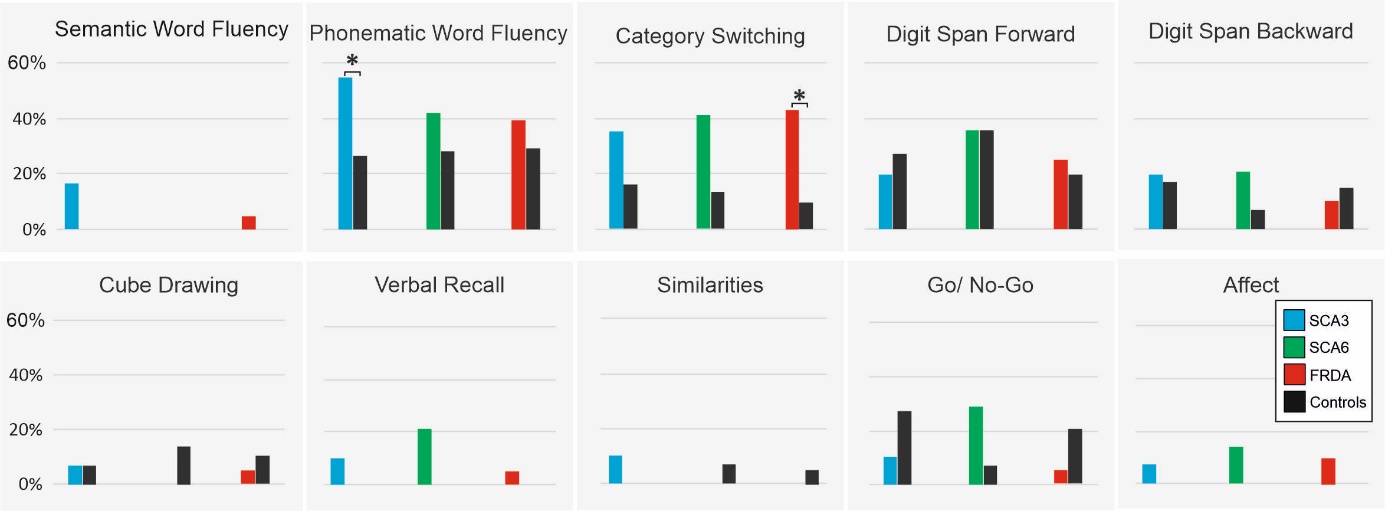


**Figure S1: Percentage of participants failing on single test items.**

Fisher’s exact test was significant (indicated by an asterisk) for phonematic word fluency in SCA3 patients and for category switching in patients with FRDA in comparison to matched controls.


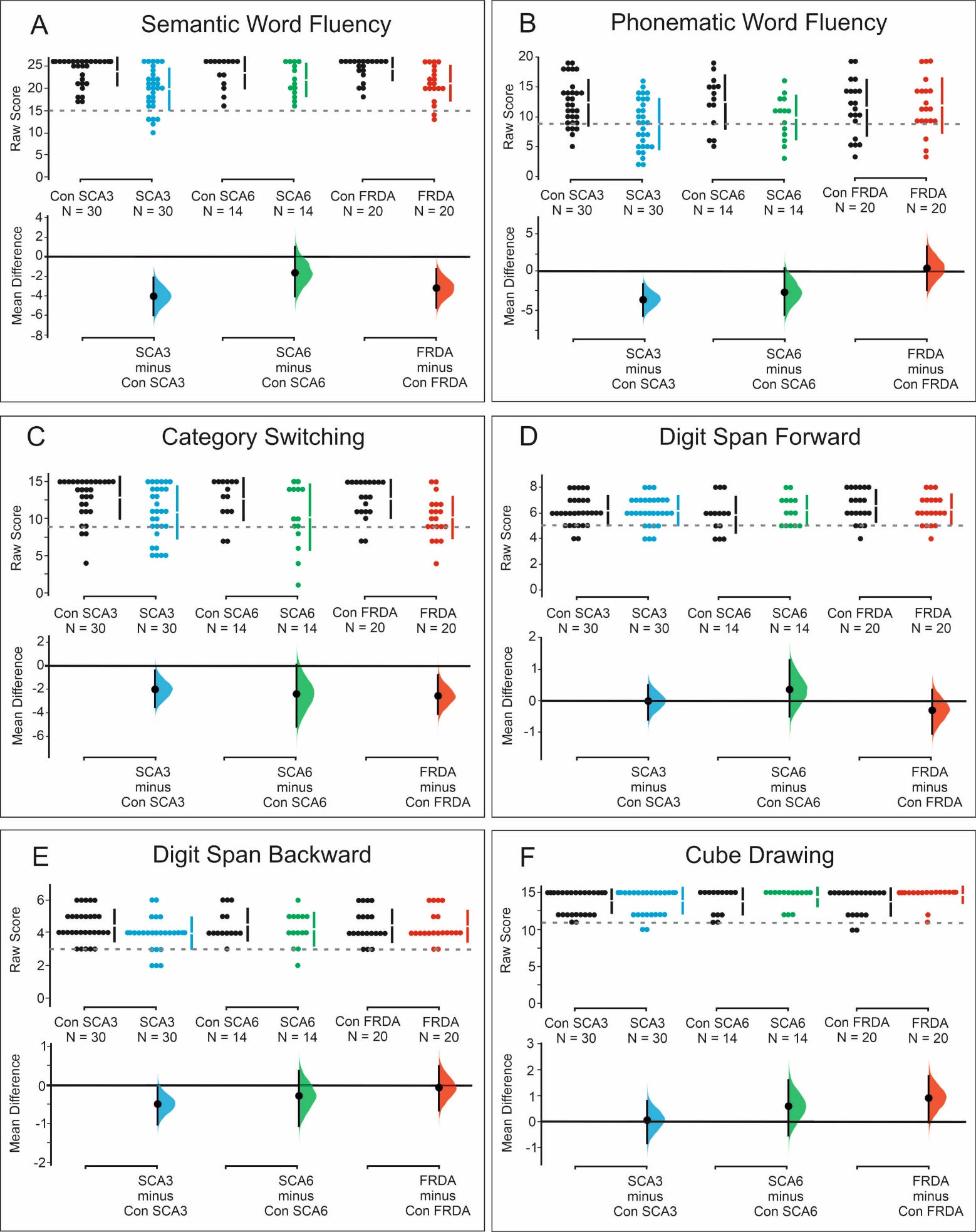


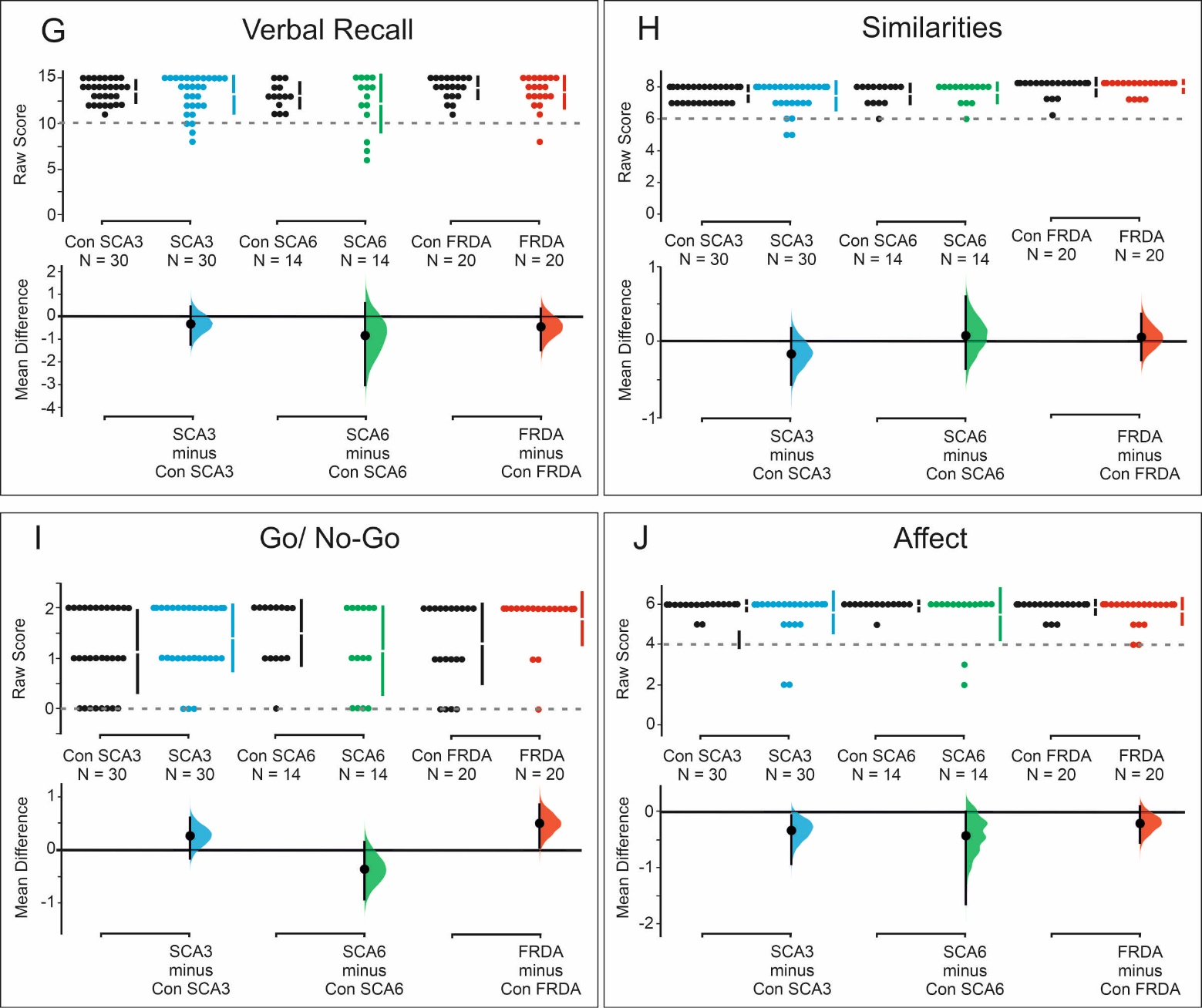


**Figure S2: Raw scores of single test items.**

The raw scores of single CCAS Scale test items are shown for SCA3 (blue), SCA6 (green) and FRDA patients (red) and their matched controls (black). **Upper panels:** Each circle represents one participant. Discontinuous bars show means and standard deviations. Dashed lines indicate the pass/fail cut-offs. **Lower panels:** Each mean difference is plotted as a bootstrap sampling distribution. Dots represent mean difference, and 95% confidence intervals are indicated by the ends of the vertical error bars.

**Table S1: Correlation analyses.**

| **SCA3** | **CCAS Scale**  **Total failed items** | **CCAS Scale**  **Total sum score** | **SARA score** | **Disease duration (yrs)** | **Age at disease onset (yrs)** | **Age (yrs)** | **Years of education** | **Repeat length longer allele** |
| --- | --- | --- | --- | --- | --- | --- | --- | --- |
| **CCAS Scale**  **Total failed items** |  | **R = -0.881**  **p < 0.001 **** | **R = 0.407**  **p = 0.026 *** | **R = 0.384**  **p = 0.036 *** | R = -0.185  p = 0.328 | R = 0.119  p = 0.530 | R = -0.212  p = 0.260 | R = 0.252  p = 0.245 |
| **CCAS Scale**  **Total sum score** |  |  | **R = -0.594**  **p = 0.001 **** | **R = -0.443**  **p = 0.014 *** | R = 0.033  p = 0.861 | R = -0.283  p = 0.129 | R = 0.253  p = 0.177 | R = -0.283  p = 0.191 |
| **SARA score** |  |  |  | **R = 0.747**  **p < 0.001 **** | R = 0.051  p = 0.788 | **R = 0.599**  **p < 0.001 **** | R = 0.092  p = 0.629 | R = 0.014  p = 0.948 |
| **Disease duration (yrs)** |  |  |  |  | R = -0.208  p = 0.271 | **R = 0.510**  **p = 0.004 **** | R = -0.097  p = 0.611 | R = -0.092  p = 0.677 |
| **Age at disease onset (yrs)** |  |  |  |  |  | **R = 0.651**  **p < 0.001 **** | R = -0.024  p = 0.9 | **R = -0.672**  **p < 0.001 **** |
| **Age (yrs)** |  |  |  |  |  |  | R = -0.208  p = 0.269 | **R = -0.632**  **p = 0.001 **** |
| **Years of education** |  |  |  |  |  |  |  | R = 0.203  p = 0.353 |

| **SCA6** | **CCAS Scale**  **Total failed items** | **CCAS Scale**  **Total sum score** | **SARA score** | **Disease duration (yrs)** | **Age at disease onset (yrs)** | **Age (yrs)** | **Years of education** | **Repeat length longer allele** |
| --- | --- | --- | --- | --- | --- | --- | --- | --- |
| **CCAS Scale**  **Total failed items** |  | **R = -0.734**  **p = 0.003 **** | R = -0.399  p = 0.177 | R = -0.308  p = 0.305 | R = -0.227  p = 0.435 | R = -0224  p = 0.441 | R = 0.097  p = 0.741 | R = 0.066  p = 0.847 |
| **CCAS Scale**  **Total sum score** |  |  | R = 0.004  p = 0.989 | R = -0.048  p = 0.875 | R = -0.062  p = 0.834 | R = -0.180  p = 0.538 | R = 0.038  p = 0.897 | R = 0.243  p = 0.472 |
| **SARA score** |  |  |  | R = 0.399  p = 0.199 | R = 0.110  p = 0.720 | R = 0.189  p = 0.537 | **R = -0.560**  **p = 0.047 *** | R = -0.161  p = 0.657 |
| **Disease duration (yrs)** |  |  |  |  | R = -0.176  p = 0.564 | R = 0.439  p = 0.133 | R = -0.290  p = 0.337 | R = 0.285  p = 0.424 |
| **Age at disease onset (yrs)** |  |  |  |  |  | **R = 0.615**  **p = 0.019 *** | R = 0.201  p = 0.490 | **R = -0.856**  **p = 0.001 **** |
| **Age (yrs)** |  |  |  |  |  |  | R = -0.078  p = 0.790 | R = -0.408  p = 0.213 |
| **Years of education** |  |  |  |  |  |  |  | R = -0.317  p = 0.342 |

| **FRDA** | **CCAS Scale**  **Total failed items** | **CCAS Scale**  **Total sum score** | **SARA score** | **Disease duration (yrs)** | **Age at disease onset (yrs)** | **Age (yrs)** | **Years of education** | **Repeat length longer allele** | **Repeat length shorter allele** |
| --- | --- | --- | --- | --- | --- | --- | --- | --- | --- |
| **CCAS Scale**  **Total failed items** |  | **R = -0.709**  **p < 0.001 **** | R = 0.268  p = 0.267 | R = 0.162  p = 0.494 | R = -0.114  p = 0.633 | R = 0.119  p = 0.617 | R = -0.107  p = 0.654 | R = 0.141  p = 0.566 | R = 0.000  p = 1.0 |
| **CCAS Scale**  **Total sum score** |  |  | R = -0.212  p = 0.384 | R = -0.231  p = 0.328 | R = -0.079  p = 0.741 | R = -0.241  p = 0.307 | R = 0.249  p = 0.290 | R = -0.036  p = 0.833 | R = 0.199  p = 0.415 |
| **SARA score** |  |  |  | **R = 0.612**  **p = 0.005 **** | R = -0.221  p = 0.363 | R = 0.213  p = 0.381 | R = -0.055  p = 0.824 | R = 0.149  p = 0.544 | R = 0.237  p = 0.328 |
| **Disease duration (yrs)** |  |  |  |  | R = 0.066  p = 0.784 | **R = 0.656**  **p = 0.002 **** | R = 0.179  p = 0.449 | R = -0.109  p = 0.658 | R = -0.167  p = 0.494 |
| **Age at disease onset (yrs)** |  |  |  |  |  | **R = 0.749**  **p < 0.001 **** | R = -0.045  p = 0.852 | **R = -0.540**  **p = 0.017 *** | **R = -0.770**  **p < 0.001 **** |
| **Age (yrs)** |  |  |  |  |  |  | R = 0.063  p = 0.793 | R = -0.319  p = 0.183 | **R = -0.653**  **p = 0.002 **** |
| **Years of education** |  |  |  |  |  |  |  | R = 0.203  p = 0.404 | R = 0.124  p = 0.613 |
| **Repeat length longer allele** |  |  |  |  |  |  |  |  | **R = 0.615**  **p = 0.005 **** |

| **All controls** | **CCAS Scale**  **Total failed items** | **CCAS Scale**  **Total sum score** | **Age (yrs)** | **Years of education** |
| --- | --- | --- | --- | --- |
| **CCAS Scale**  **Total failed items** |  | **R = -0.658**  **p < 0.001 **** | **R = 0.320**  **p = 0.01 **** | R = -0.246  p = 0.050 |
| **CCAS Scale**  **Total sum score** |  |  | **R = -0.346**  **p = 0.005 **** | R = 0.237  p = 0.059 |
| **Age (yrs)** |  |  |  | R = -0.073  p = 0.566 |

Spearman’s rank correlation coefficient (R) and p-values are given for all correlations. Significant results are indicated by asterisks (* for a significance
 level of p < 0.05 and ** for a level of < 0.005). Abbreviations: SCA3 = spinocerebellar ataxia type 3, SCA6 = spinocerebellar ataxia type 6, FRDA
 Friedreich ataxia, CCAS-Scale = Cerebellar Cognitive Affective Syndrome Scale, SARA = Scale for the Assessment and Rating of Ataxia, yrs = years.


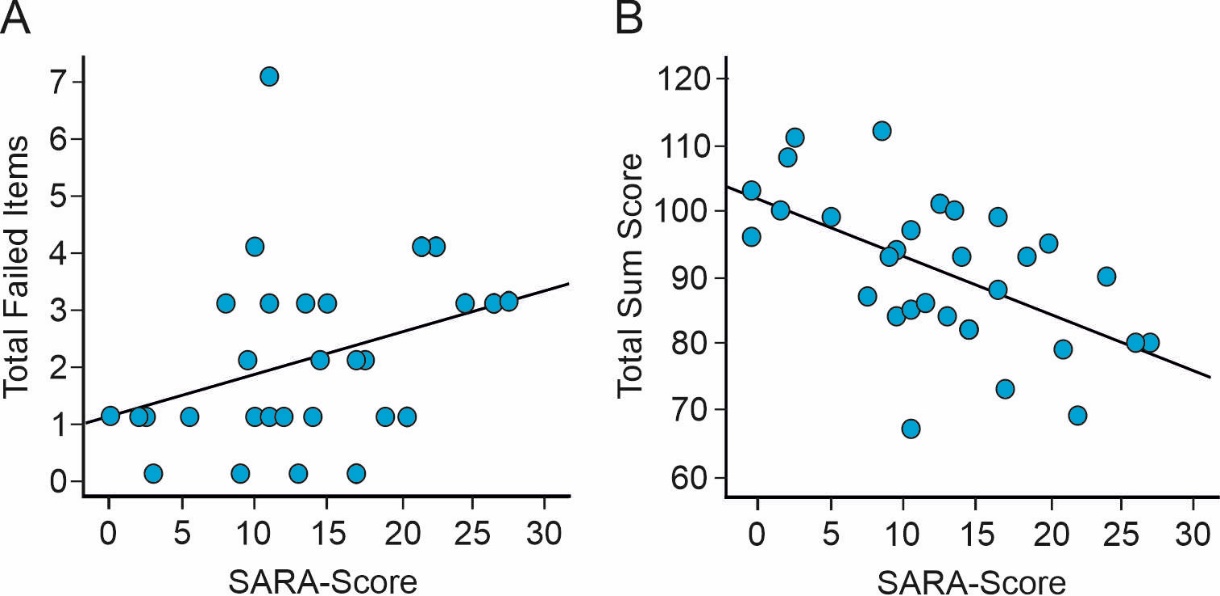


**Figure S3: Correlations between total failed test items (A) and total sum score (B) and SARA score in SCA3 patients.**
